# *BMC Rheumatology*: a home for all rheumatology research within the BMC Series

**DOI:** 10.1186/s41927-017-0006-3

**Published:** 2017-11-28

**Authors:** James Mockridge, Alessia Alunno, Susan Goodman, Edward Roddy

**Affiliations:** 1 BioMed Central, 236 Gray’s Inn Road, London, WC1X 8HB UK; 2 Rheumatology Unit, Department of Medicine, University of Perugia, Piazzale Menghini, 06129 Perugia, Italy; 3 Hospital for Special Surgery, 535 East 70th Street, New York, NY 10021 USA; 4 Arthritis Research UK Primary Care Centre, Research Institute for Primary Care and Health Sciences, Keele University, Keele, Staffordshire ST5 5BG UK

## Abstract

This editorial accompanies the launch of *BMC Rheumatology*, a new open access, peer-reviewed journal within the BMC Series portfolio, which considers studies on all aspects of rheumatological diseases. The Journal will also place a special emphasis on manuscripts reporting systemic and inflammatory conditions and connective tissue diseases, along with related comorbidities, including cardiovascular disease, malignancy and infection. Through the publication of a variety of article types, including Research articles, Case reports, Study protocols and Debates, *BMC Rheumatology* will provide the rheumatology community with an open access avenue to disseminate research into rheumatological diseases, with the ultimate aim of improving patient care.

**Electronic supplementary material:**

The online version of this article (10.1186/s41927-017-0006-3) contains supplementary material, which is available to authorized users.

## Introduction

Rheumatology is a multidisciplinary branch of medicine concerned with the study and treatment of a wide variety of disorders collectively known as rheumatological diseases. Many of these disorders affect the joints, muscles and bones, and cause swelling, stiffness, pain and deformity; however, the term rheumatological disease extends beyond the musculoskeletal system itself and includes a wide variety of related systemic and inflammatory conditions, and heritable connective tissue disorders, and thus affecting multiple organ systems and tissues. Nevertheless, as these disorders generally involve inflammation or the involvement of the immune system (auto-immunity), rheumatology is generally seen increasingly moving in the direction of autoimmunity.

Rheumatological diseases have a significant burden and impact on healthcare systems and are one of the most common causes of disability and work-related absences. For example, in the UK alone, approximately 9 million people are affected by osteoarthritis and rheumatoid arthritis (RA), conditions which affect the knees, hips and hands/wrists [1]. Gout, which is caused by the build-up of monosodium urate crystals in the joints and results in painful joint inflammation, has been reported to have a prevalence of 2.49% of the population in the UK, equivalent to approximately 1.5 million people [2]. Aside from the most common rheumatological diseases, there are a further 100 or so disorders encompassing the umbrella term, many of which are systemic in nature, and include less common disorders such as giant cell arteritis (GCA), polymyalgia rheumatica (PMR), myositis, systemic lupus erythematosus (SLE), fibromyalgia, Sjögren’s syndrome, and scleroderma to name a few.

In addition to the disease burden, patients with rheumatological diseases have been found to be at an increased risk of developing a variety of comorbid conditions. There is a growing list of comorbidities that are frequently associated with rheumatological diseases and these include malignancies, cardiovascular diseases, infections, kidney diseases, lung diseases, gastrointestinal diseases, osteoporosis and depression. These comorbidities have a significant effect on the lives of patients and therefore it is important to increase our understanding of these conditions to ensure optimal treatment and improvements in quality of life.

It is upon this background that *BMC Rheumatology* is being launched to provide the rheumatology community with an open access avenue to disseminate research findings across the range of rheumatological diseases and their associated comorbidities, with the ultimate aim of improving patient care.

## Aims and scope

*BMC Rheumatology* considers research on all aspects of the prevention, treatment and management of rheumatological diseases, and will place a special emphasis on systemic and inflammatory conditions and connective tissue diseases, along with related comorbidities. The Journal will publish a variety of article types, including Research articles, Case reports, Debates and Database articles, as well as Study protocols for proposed or on-going prospective clinical research.

*BMC Rheumatology* will complement and expand the open access journal portfolio within the area of rheumatology at BioMed Central and will be a sister journal to *BMC Musculoskeletal Disorders* within the BMC Series. Although there is some overlap in scope, *BMC Rheumatology* and *BMC Musculoskeletal Disorders* sit very comfortably alongside each other: *BMC Musculoskeletal Disorders* has a focus on studies with a definite musculoskeletal component (i.e. bone, cartilage, tendon etc.), whereas *BMC Rheumatology* will cover all areas of rheumatology, including systemic conditions and related comorbidities, which are topics that fall outside the scope of *BMC Musculoskeletal Disorders*.

As part of the BMC Series, which is a collection of high-quality, peer-reviewed journals covering all areas of biology and medicine and focusing on the needs of the research communities which they serve, the Journal does not make editorial decisions on the basis of the interest of a study or its likely impact; however, studies must be scientifically valid. For research articles this includes a scientifically sound research question, the use of suitable methods and analysis, and following community-agreed standards relevant to the research field.

*BMC Rheumatology* has three editorial sections:

- Clinical rheumatology
- Epidemiology and public health
- Pre-clinical and translational science

We are delighted to welcome Susan Goodman, Edward Roddy and Alessia Alunno as Section Editors for the Journal, along with an expanding international team of Associate Editors [3]. Together with the in-house Editor, they will provide academic leadership and expertise. As the Journal grows and develops, we will continue to recruit academic editors to the Editorial Board in order to adapt to the changing and growing nature of the field.

In order to ensure the transparency and fairness of the review process and to give credit to reviewers for their efforts, *BMC Rheumatology*, like all of the medical journals in the BMC Series, operates an open peer review system. Open peer review means that the identity of the peer reviewers is disclosed and their names are included on the peer review reports, so that authors can see who has reviewed their manuscript. Once the article has been published, the reviewer reports throughout the peer review process and corresponding author responses are made available online along with the final version of the manuscript. The online published article will provide a link to its ‘pre-publication history’, which lists all of the versions of the manuscript, all the signed reviews, and all responses to the reviewers from the submission of the manuscript to its publication.

*BMC Rheumatology* aims to publish work deemed by peer reviewers to be a coherent and valid addition to scientific knowledge, and to provide an all-important open access platform to allow the effective dissemination of this knowledge, so that we can all work together to understand more fully and explore the most important developments, trends, and practices in rheumatological diseases and their associated comorbidities. We believe that open access and the Creative Commons Attribution License [4] is essential in this, allowing universal and free access to all articles published in the Journal and allowing them to be read and the data re-used without any restrictions. *BMC Rheumatology* will work closely with the other journals in the BMC Series portfolio [5] to help authors find the right home for their research in the field of rheumatology.

## Clinical rheumatology section

The clinical rheumatology section has a broad portfolio: our new journal intends to address all aspects of clinical rheumatology. We aim to publish papers from all areas regarding those rheumatological diseases that are systemic, rather than the mechanical or regional musculoskeletal syndromes. The rapid advances in diagnosis, management, and therapy in the rheumatological diseases have fostered the need for more focused publications and has facilitated our separation from *BMC Musculoskeletal Disorders.*

There has been an explosion of publications defining disease diagnosis and measurement for patients with RA and spondyloarthritis (SpA), and the application of aggressive management strategies mandates careful assessment of disease status and response to interventions. Careful determination of disease subsets to predict treatment responses can avoid unnecessary drug exposure and avoid delay when drug exposure is warranted requires a forum for disseminating information. The gratifying expansion of effective treatment options has built on the precise definition of measurement strategies so that the application and response to treatment can be assessed. Do we know which patient with SpA should receive a non-steroidal anti-inflammatory drug (NSAID) alone vs a biologic? How do we know when to taper medications in inflammatory arthritis or if it is safe to discontinue drug therapy? We hope to publish the investigations as well as the in-depth expert reviews to help our readers answer these questions as knowledge advances.

The promising therapeutic benefit of interleukin-6 (IL-6) inhibition with tocilizumab for patients with PMR/GCA has created a need for new strategies to accelerate diagnosis and improve disease assessments. Diagnostic modalities will likely evolve along with treatment strategies; we will need to understand the role of imaging in early assessment. Do we know the best way to prevent complications of GCA such as loss of vision, and do we know when it is safe to taper or discontinue tocilizumab? These advances have created the need for publications to reliably facilitate dissemination as well as assessment of information as it becomes available.

Treatment breakthroughs have been more elusive for scleroderma (systemic sclerosis) and Sjögren’s syndrome, highlighting the unmet needs for better understanding of the pathogenesis of these diseases so that treatment targets can be developed. For patients with SLE, the recent development of belimumab as SLE-specific therapy leads to thought about the limited understanding of therapeutic targets in SLE and the need for better understanding of SLE epidemiology and pathogenesis. Progress can be enhanced by the rapid and open dissemination of well-designed trials and observational studies, as well as expert’s views to contextualize studies. *BMC Rheumatology* aims to provide a new platform to help assess the explosion of rheumatology information.

## Epidemiology and public health section

Epidemiology is defined as the study of the occurrence and distribution of health-related events, states, and processes in specified populations, including the study of the determinants influencing such processes, and the application of this knowledge to control relevant health problems [6]. Epidemiological methods are varied and can be applied to answer many different questions in clinical and health-related research such as quantifying the prevalence and incidence of disease and how these change over time; describing and assessing specific features and characteristics of disease; identifying risk factors for and complications of diseases; and describing natural history and prognosis. There is a long history of excellent epidemiological research in rheumatology across the globe which has made major contributions to our understanding of the burden of rheumatological diseases and their causation and outcome. Selected examples of the many noteworthy epidemiological research studies of relevance to rheumatology include:

- contrasting trends in disease occurrence between rheumatological conditions. The incidence of gout is rising reflecting trends in the population prevalences of obesity, adverse lifestyles and comorbidity [2], whereas systemic rheumatoid vasculitis appears to be becoming less common, probably as a result of more effective treatments for RA [7];
- careful clinical observations leading to the historical recognition of new diseases such as the first descriptions of nodal osteoarthritis and psoriatic arthritis [8, 9];
- prospective cohort studies describing the increased risk of lymphoproliferative disease in RA [10] and premature cardiovascular disease in SLE [11];
- surveillance of adverse effects of treatment in routine clinical practice through drug registries, such as the British Society for Rheumatology Biologics Register [12, 13].

The epidemiology and public health section of *BMC Rheumatology* aims to create a platform through which to disseminate examples of continued excellence in this long tradition of rheumatological epidemiology. We aim to publish original research articles relating to all aspects of the epidemiology of rheumatological diseases, related systemic and inflammatory conditions and comorbidity, including but not limited to studies of disease occurrence, risk factors, outcomes and prognosis, and encompassing a wide range of study designs and methodologies.

## Pre-clinical and translational science section

Rheumatology has witnessed rapid advances over the last few decades and translational science is gaining increasing prominence [14]. By pursuing the “bench to bedside” approach to rapidly implement the newest research findings into patient care, translational research in rheumatology aims at closing the gap of personalized medicine and provide an answer to the longstanding question of who to treat, when, with what and for how long. Novel technologies allow us to redefine rheumatological diseases at a molecular level and reveal disease-associated pathways. Genome wide association studies (GWAS) have identified many genes linked with disease predisposition and evolution while an increasing number of studies also support the pivotal role of the microbiome in the multistep process of RA pathogenesis, with *P. gingivalis* being the most prominent example. Furthermore, in the era of the ‘omics’ platforms and next-generation sequencing, researchers can access a massive amount of information about the molecular signature of rheumatological disease. All these exciting developments open new research avenues to ideally reach the appealing goal of rheumatological disease prevention. However, also old techniques, such as immunohistochemistry historically employed only for diagnostic purposes, have been recently acknowledged as potential prognostic tools in diseases such as RA and Sjögren’s syndrome boosting research studies to possibly predict disease evolution and response to treatment by integrating experimental results and patient health record.

The pre-clinical and translational science section of *BMC Rheumatology* aims to publish papers focused on the identification of the molecular underpinnings of the disease and the application of this knowledge for the discovery of new drug targets or for patient stratification. We aim to publish original research articles relating to all aspects of pre-clinical and translational research in rheumatology which covers a wide range of methodologies and study designs.

## Conclusion

*BMC Rheumatology* is being launched to provide a community-driven, authoritative, unbiased and fully open access journal publishing all aspects of research into rheumatological diseases, and to provide a home for all scientifically valid manuscripts in this exciting research area. We are looking forward to working closely with authors, reviewers and members of the Editorial Board to provide the community with high-quality research into this diverse medical speciality.

We hope you will find the first articles published in the Journal an interesting and valuable read, and we look forward to working with you all to disseminate research into rheumatological diseases.

## Additional file

Additional file 1: Reviewer reports and AU response to reviewers. (DOCX 15 kb)

## Abbreviations

GCA: Giant cell arteritis
PMR: Polymyalgia rheumatic
RA: Rheumatoid arthritis
SLE: Systemic lupus erythematosus
SpA: Spondyloarthritis
