# Supplementary material for: BMC Rheumatology: a home for all rheumatology research within the BMC Series
Source: BMC Rheumatol. 2017 Nov 28;1:1. doi: 10.1186/s41927-017-0006-3 (PMC6383591; doi:10.1186/s41927-017-0006-3)
Supplement: Supplementary file 1 — Reviewer reports and AU response to reviewers. (DOCX 15 kb) [file 41927_2017_6_MOESM1_ESM.docx]

**Open Peer Review summary**

Pre-publication versions of this article, reviewer reports and author comments to reviewers are available by contacting [info@biomedcentral.com](mailto:info@biomedcentral.com).

| Original submission | | |
| --- | --- | --- |
| 26 October 2017 | Submitted | Original manuscript |
| Publishing |  |  |
| 30 October 2017 | Editorially accepted |  |
| 28 November 2017 | Article published | 10.1186/s41927-017-0006-3 |

### How does Open Peer Review work?

Open peer review is a system where authors know who the reviewers are, and the reviewers know who the authors are. If the manuscript is accepted, the named reviewer reports are published alongside the article. Pre-publication versions of the article are available by contacting [info@biomedcentral.com](mailto:%20mailto:info@biomedcentral.com).

You can find further information about the peer review system at <https://bmcrheumatol.biomedcentral.com/about#peer-review+policy>
